# *Lactobacillus casei* BL23 Produces Microvesicles Carrying Proteins That Have Been Associated with Its Probiotic Effect

**DOI:** 10.3389/fmicb.2017.01783

**Published:** 2017-09-20

**Authors:** A. Paula Domínguez Rubio, Jimena H. Martínez, Diana C. Martínez Casillas, Federico Coluccio Leskow, Mariana Piuri, Oscar E. Pérez

**Affiliations:** ^1^Departamento de Química Biológica, Facultad de Ciencias Exactas y Naturales, Universidad de Buenos Aires Buenos Aires, Argentina; ^2^Instituto de Química Biológica de la Facultad de Ciencias Exactas y Naturales, Universidad de Buenos Aires, Consejo Nacional de Investigaciones Científicas y Técnicas Buenos Aires, Argentina; ^3^Departamento de Física de la Materia Condensada, Centro Nacional de Energía Atómica Buenos Aires, Argentina; ^4^Departamento de Ciencias Básicas, Universidad Nacional de Luján Buenos Aires, Argentina; ^5^Departamento de Desarrollo Productivo y Tecnológico, Universidad Nacional de Lanús Buenos Aires, Argentina

**Keywords:** microvesicles, *Lactobacillus casei* BL23, probiotics, vesicle size distribution, CFSE, proteomics

## Abstract

Archaea, bacteria, and eukarya secrete membrane microvesicles (MVs) as a mechanism for intercellular communication. We report the isolation and characterization of MVs from the probiotic strain *Lactobacillus casei* BL23. MVs were characterized using analytical high performance techniques, DLS, AFM and TEM. Similar to what has been described for other Gram-positive bacteria, MVs were on the nanometric size range (30–50 nm). MVs carried cytoplasmic components such as DNA, RNA and proteins. Using a proteomic approach (LC-MS), we identified a total of 103 proteins; 13 exclusively present in the MVs. The MVs content included cell envelope associated and secretory proteins, heat and cold shock proteins, several metabolic enzymes, proteases, structural components of the ribosome, membrane transporters, cell wall-associated hydrolases and phage related proteins. In particular, we identified proteins described as mediators of *Lactobacillus’* probiotic effects such as p40, p75 and the product of LCABL_31160, annotated as an adhesion protein. The presence of these proteins suggests a role for the MVs in the bacteria-gastrointestinal cells interface. The expression and further encapsulation of proteins into MVs of GRAS (Generally Recognized as Safe) bacteria could represent a scientific novelty, with applications in food, nutraceuticals and clinical therapies.

## Introduction

Functional foods benefit human health beyond their basic nutritional properties (Ferguson, 2009). They are consumed in a normal diet and contain biologically active components that can offer health benefits and reduce the risk of disease. Micronutrients, vitamins and minerals are well-established functional ingredients. Probiotics together with prebiotics, lipids and phytonutrients belong to the new generation of active ingredients (Jankovic et al., 2010). Probiotics are live microorganisms which, when administered in adequate amounts, confer a health benefit on the host (Hill et al., 2014). They are included in cheese, yogurts and fermented milks, or available as dietary supplements in the form of a dehydrated product (Stanton et al., 2001).

Research conducted on probiotics over the past 25 years has supported the beneficial effects of probiotics (Jankovic et al., 2010). They have been associated with the treatment or prevention of allergic diseases (Prescott et al., 2007) as well as with a positive effect in the regulation of endocrine, nervous, circulatory and digestive system in humans (O’Mahony et al., 2005; Benton et al., 2007; Rincón et al., 2014; Mayer et al., 2015). The popularity of probiotic use has increased dramatically in the last decades, blossoming into a $25 billion per year global industry, with widespread use, not only in clinical care, but also in healthy individuals wishing to maintain a healthy gut microbiome (Fleming et al., 2016; Islam, 2016). The turnover value of the global probiotics market is projected to reach a value of US$46.55 billion by 2020. This market is dominated by probiotics producing companies, nutritional supplements and food companies (O’Toole et al., 2017).

There is a wide variety of genera and species of microorganisms as potential probiotics, being the most commonly used the genera *Lactobacillus* and *Bifidobacterium* (Ross et al., 2005). These Gram-positive bacteria are regular residents of the mammalian gastrointestinal microbiome and have long been used in food fermentations, being awarded the status of “Generally Recognized As Safe” (GRAS) (Dunne et al., 2001). Probiotics must survive food processing or product maturation and shelf-life for successful delivery in foods. A wide variety of species of the genus *Lactobacillus* are technologically more fitted for food applications than *Bifidobacterium* (Ross et al., 2005; Lee and Salminen, 2008). Moreover, due to the capacity to survive in the gastrointestinal tract and the adhesion to the intestine, *Lactobacillus* have emerged as a mucosal delivery system being a potential alternative to others, such as nanoparticles, liposomes, microspheres, immunomodulating complexes, and attenuated pathogens (Wang et al., 2016; Kuczkowska et al., 2017).

Extracellular vesicles are a form of intracellular and extracellular communication used by archae, bacteria and eukaryotes (Deatheragea and Cooksona, 2012; Al-Nedawi et al., 2014; Yáñez-Mó et al., 2015; Pathirana and Kaparakis-Liaskos, 2016). Extracellular vesicles are spherical bilayered membrane sacs. Production of membrane vesicles has been reported in Gram-positive bacteria and despite its nanometric size, between 10 and 400 nm, they are known as microvesicles (MVs) (Brown et al., 2014). They contain cytoplasmic components such as DNA, RNA and proteins. The conditions modulating MVs formation remain elusive, although some reports showed that they are constantly shedding from bacteria (Brown et al., 2014). So far, little is known regarding their production and their cargo.

Concerning to the genus *Lactobacillus*, MVs production has only been described for *L. rhamnosus* (JB-1) and *L. plantarum* (Al-Nedawi et al., 2014; Li et al., 2017). Immune regulation by MVs from lactic acid bacteria has been proposed to be involved in signaling between probiotic intestinal bacteria and their mammalian hosts (Al-Nedawi et al., 2014). On the other hand, *L. casei* BL23 is an extensively studied model strain (Watterlot et al., 2010; Bermúdez-Humarán et al., 2011) proposed as probiotic due to its anti-inflammatory effects (Rochat et al., 2007) and its ability to prevent experimental colitis in mouse models (Foligne et al., 2007). Additionally, *L. casei* BL23 orally inoculated mice have shown a decrease of *Listeria monocytogenes* systemic dissemination (Archambaud et al., 2012) and a modulation of the host immune response protecting mice against induced colorectal cancer (Lenoir et al., 2016).

In the present study we report the isolation of MVs from *L. casei* BL23. MV cargo and morphology were also characterized. To our knowledge, there are no previous reports of MVs production by this probiotic strain. We analyzed MV particle size, surface charge, topography and microstructural morphology, and their nucleic acid and protein contents were quantified including a detailed proteomic analysis by LC-MS.

## Materials and Methods

### Cultures and Microvesicles Isolation

**FIGURE 1:**
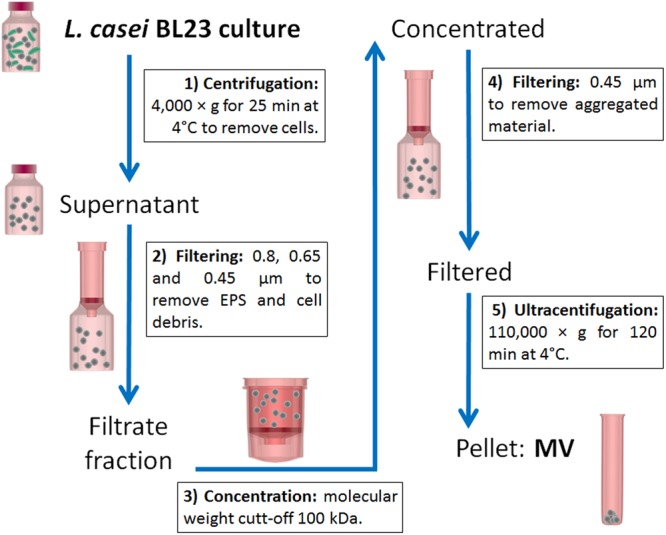
Schematic representation of the protocol used to isolate *L. casei* BL23 MVs. Cells were removed from the culture by centrifugation and subsequent filtration through a series of decreasing pore membranes: 0.8, 0.65, and 0.45 μm. Cell free supernatant was then concentrated using a 100 kDa filter membrane. Finally, the concentrated supernatant was spun at 110,000 *g* to pellet MVs while the soluble proteins remained in the supernatant.

*Lactobacillus casei* BL23 strain was a kind gift of Dr. Gaspar Pérez Martínez (Instituto de Agroquímica y Tecnología de Alimentos, Valencia, Spain). *L. casei* BL23 was grown in Man-Rogosa-Sharpe (MRS, Biokar Diagnostics) medium at 37°C for 48 h (800 ml). MVs were isolated according to the protocol represented in **Figure 1** which is a modified method of that proposed by Rivera et al. (2010) and Pospichalova et al. (2015).

Cultures were spun at 4,000 × g for 25 min at 4°C to remove cells. The resulting supernatant was then filtered through a series of decreasing pore-size membranes: 0.8, 0.65, and 0.45 μm (GE Osmonics membranes, Lenntech) to remove exopolysaccharide (EPS) and cellular debris. Concentration of the filtrate fraction was done using a Centricon ultrafiltration system with 100 kDa cut-off filter (Sartorius). The concentrate was subjected to further filtration through a 0.45 μm filter for removing of aggregated material. The filtered supernatant was then centrifuged at 110,000 × g for 120 min at 4°C in a SW 41 Ti rotor (Beckman Optima L-80, Beckman Coulter, United States) and washed with phosphate buffered saline solution (PBS). The pellet containing the vesicles was resuspended in PBS or Quick-Zol reagent (Kalium Technologies) and finally stored at -80°C. Concentrated MVs were stored under those conditions until their use.

MRS without added bacteria was used as negative control. *Bacillus subtilis* 168, was obtained from the *Bacillus* Genetic Stock Center and grown in brain heart infusion (BHI, Merck Millipore) medium at 37°C under continuous agitation at 200 rpm for 18 h (100 ml). This species was used as positive control because of its high production capacity of MVs (Brown et al., 2014; Kim et al., 2016).

### Microstructural Analysis of MVs

#### Size Determinations

Dynamic light scattering experiments were carried out in a dynamic laser light scattering (DLS) (Zetasizer Nano-Zs, Malvern Instruments, United Kingdom) with a measurement range of 0.6 nm to 6 μm, provided with a He-Ne laser (633 nm) and a digital correlator Model ZEN3600. Measurements were achieved at a fixed scattering angle of 173°. Samples were in a disposable polystyrene cuvette. The sample is illuminated with a laser beam and the intensity of the resulting scattered light produced by the particles fluctuates at a rate that is dependent upon the size of the particles. Analysis of these intensity fluctuations yields the diffusion coefficient of the particle and therefore the particle size using de Stokes-Einstein equation:

(1) $$\begin{array}{c} d(H)\;=\;kT/6\;\pi \;\eta D \end{array}$$

where, d(H): hydrodynamic diameter; D: translational diffusion coefficient; k: Boltzmann’s constant; T: absolute temperature; η: viscosity.

CONTIN method was used to obtain size information by mean the data concerning to percentile distribution of particle or aggregate sizes. This size distribution is a plot of the relative intensity of light scattered by particles in various size classes and it is known as an *intensity* size distribution. Using Mie theory, it is possible to convert the intensity distribution to *volume* distribution, that is important to analyze the relative significance of each peak. Since the peaks of higher size generate greater intensity than the smaller ones, the intensity is proportional to the square of the molecular weight. The assay was performed on quadruple independently isolated samples. Polydispersity Index (PDI) is also informed. The PDI is dimensionless and scaled such that values lower than 0.05 are rarely observed other than with highly monodisperse standards. Values greater than 0.7 suggest that the sample has a very broad size distribution and is probably not appropriate for the this technique (Instruments Malvern, 2011). We determined the Z- average, which represents the mean diameter of the particles, and it is beneficial when comparing one average value with a monomodal distribution, but clearly inadequate for describing distribution in polydisperse systems (Camino et al., 2009).

#### Surface Charge Measurements

Surface charge measurements were evaluated as ζ-potential measurements and also performed in a dynamic DLS instrument (Zetasizer Nano–Zs, Malvern Instruments, United Kingdom). The ζ-potential was evaluated from the electrophoretic mobility of the particles. The processing of the measured electrophoretic mobility data into ζ–potential was done using Henry’s equation

(2) $$\begin{array}{c} \mathrm{Ue}\;=\;2\varepsilon \zeta \;f(\mathrm{Ka})/3\eta \end{array}$$

where Ue is the electrophoretic mobility, 𝜀 the dielectric constant, η the sample viscosity and *f*(Ka) the Henry’s function (Pérez et al., 2014).

The reported values correspond to the average and standard deviation from measurements of each of three MV isolation process.

#### Atomic Force Microscopy

The topography corresponding to MVs was obtained by atomic force microscopy (AFM) analysis. It was performed under tapping mode AFM (Nanoscope IIIa, Veeco, United States) applying special TM AFM tips (model RTESP-300 Bruker) with nominal radius of curvature 10 nm. A typical force constant of 21.84 N/m, and resonant frequency of 250 kHz was applied. MVs from *L. casei* BL23 and *B. subtilis* 168 were seeded onto cleaved glass. The surfaces were dried under a sterile air-flow cabinet for 30 min at room temperature. Both topography and shaded topography images were recorded using NanoScope software. Topography images were processed with Gwyddion software (Czech Metrology Institute) and NanoScope Analysis (Veeco) software (Abuin et al., 2010).

#### Transmission Electron Microscopy

Isolated MVs from *L. casei* BL23 were placed onto a grid for 10 min and mixed with an equal quantity of 2% aqueous solution of uranylacetate for 3 min. The mixture was washed in distilled water and then the surface was dried under a sterile air-flow cabinet at room temperature. MVs were observed under a Zeiss EM 109-T appliance (Zeiss, Germany) and images were obtained with a coupled Gatan ES1000W CCD camera (Durante et al., 2015). Image processing was performed with ImageJ software (NIH).

### Confocal Laser Scanning Microscopy

Lactobacillus casei BL23 cells were stained with carboxyfluorescein succinimidyl ester (CFSE) (Invitrogen) at 10 μM final concentration in PBS for 30 min at 37°C. The bacteria were washed three times in PBS at 4000 g for 5 min at 4°C. Labeled bacteria were fixed with paraformaldehyde (4% prepared in PBS) for 30 min at room temperature. Slides were coated with poly-D-lysine (100 μg/ml) for 1 h at 37°C. Labeled bacteria were mounting with Mowiol and examined by confocal laser scanning microscopy using an Olympus FV 1000 module (Olympus, Japan). Fluorescent images consisted of 1024 × 1024 pixels and were taken at PLAPON 60X water objective NA = 1.42 (1 μm = 19.3 pixel). Isolated MVs were stained with CFSE with the same protocol.

CFSE has a peak excitation of 494 nm and peak emission of 521 nm, which is measured using 488 nm laser excitation (blue) and 535/35 band pass filter for detection (green) (Pospichalova et al., 2015). Confocal laser scanning microscopy images are representative of three independent experiments. Images were taken of at least ten fields of view and processing with FIJI (ImageJ) software (NIH).

### Biochemical Analysis

#### DNA, RNA and Protein Quantification

Total DNA, RNA and protein were isolated from L. casei BL23 microvesicles using 1 ml of Quick-Zol (Kalium Technologies) reagent following the manufacturer’s instructions. DNA and RNA were quantified by UV absorbance using a NanoDrop 2000/2000c (Thermo Fisher Scientific, United States). The protein content was quantified by Lowry’s method (Lowry et al., 1951). The reported values correspond to the average and standard deviation of three measurements.

#### SDS Polyacrylamide Gel Electrophoresis and Proteomic Analysis

Proteins were separated on a SDS-PAGE gel (12% resolving gel) followed by Coomassie blue staining (*n* = 5). Extracted proteins of MVs and bacteria were digested with trypsin and analyzed by nano-HPLC coupled to mass spectrometry with Orbitrap technology (LC-MS) (Wada et al., 2014). The protein spectrum was analyzed against *L. casei* BL23 proteome obtained from the genome sequence (NC_010999.1) (Mazé et al., 2010). Data were filtered using the Proteome Discoverer 2.1 software to obtain maximum protein and peptide false discovery rate of 1% calculated by employing a reverse database strategy. Raw data was deposited in EVpedia (evpedia.info/) (Kim et al., 2015).

## Results

### Isolation of *L. casei* BL23 MVs

**FIGURE 2:**
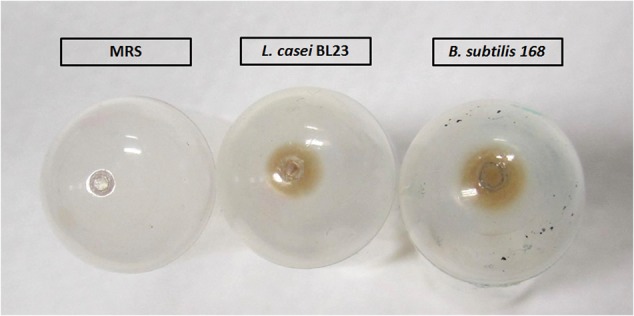
Macroscopic aspect of *L. casei* BL23 and *B. subtilis* 168 pellets containing MVs.

MVs from *L. casei* BL23 and *B. subtilis* 168, used as positive control, were isolated from liquid cultures according to the protocol described in Section “Materials and Methods” (**Figure 1**). The aspect, morphology and color of the pellet containing the MVs resulted different for both species. Concentration of *B. subtilis* 168 culture supernatants produced a bigger brown vesicle pellet, whereas processing of *L. casei* BL23 culture supernatant resulted in a smaller and lighter pellet. As expected, no pellet was observed in a negative control using MRS media alone (**Figure 2**). MVs have been purified from Gram-positive bacteria during the late exponential or stationary growth phases (Kim et al., 2016). In this study, MVs were collected from stationary phase cultures. Moreover, concentrated supernatants from *L. casei* BL23 at 24 h (early stationary phase) produced a smaller MV pellet in comparison to 48 h (late stationary phase) (data not shown), so for further characterization the MVs were collected at this later time point.

### Microstructural Analysis

#### Size Characterization of *L. casei* BL23 MVs

**FIGURE 3:**
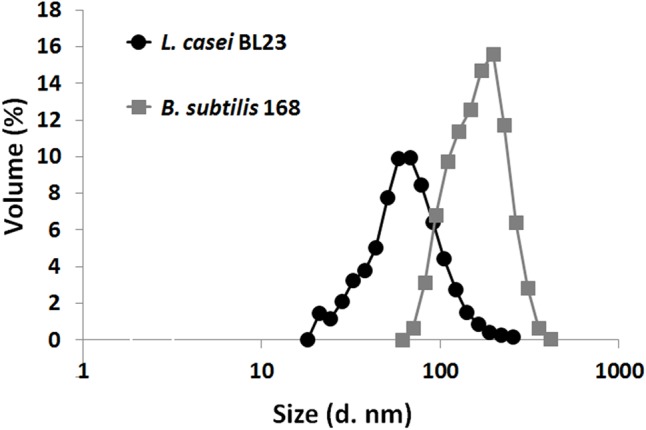
Particle size distribution curves of *L. casei* BL23 and *B. subtilis* MVs as determined based on DLS data. Each point represents the mean ± SD of independently isolation processes (*n* = 4).

Particle size distribution of isolated MVs from *L. casei* BL23 and *B. subtilis* 168 cultures was studied comparatively using DLS (**Figure 3**). DLS revealed one population, i.e., monomodal distribution, with a peak between 26 and 70 nm for MVs obtained from *L. casei* BL23 (**Table 1**). On the other side, *B. subtilis* 168 MVs particle size also revealed only one population of approximately 71 and 245 nm, larger than those of *L. casei* BL23 (**Table 1**).

**Table 1:** ζ-potential (mean ± SEM), PDI (polydispersity index) and diameter measured by DLS, AFM, and TEM (mean ± SEM) (*n* = 4).

	ζ-potential (mV)	PDI	Diameter DLS (nm)	Diameter AFM (nm)	Diameter TEM (nm)
*L. casei* BL23	–8.7 ± 1,9	0.38 ± 0.04	47 ± 3	33 ± 3	48 ± 3
*B. subtilis* 168	–18.2 ± 1,7	0.61 ± 0.06	142 ± 14	310 ± 5	52 ± 3

#### MV’s Membrane Potential

To gain insight into the stability of the MV particles in terms of aggregation, flocculation or dispersion, we measured the zeta potential of MVs. Dynamic light scattering can measure the MVs surface charge as represented by the ζ-potential (Sokolova et al., 2011). The ζ-potential determination of MV’s preparations from *L. casei* BL23 and *B. subtilis* 168 revealed negative values in PBS (pH: 7.4) for both species at 25°C (**Table 1**).

#### Atomic Force Microscopy

**FIGURE 4:**
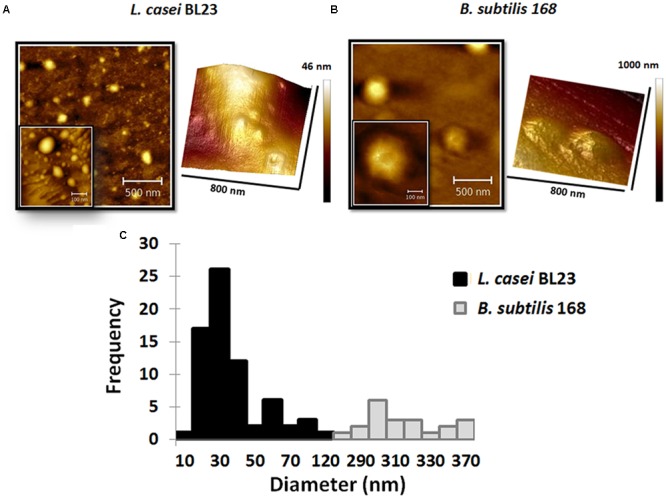
Atomic force microscopy (AFM) topography of MVs. Phase images and 3D reconstruction of MVs of *L. casei* BL23 **(A)** and *B. subtilis* 168 **(B)**. Size-frequency measurements of MVs expressed as a histogram **(C)**.

In atomic force microscopy a mechanical cantilever is passed over a surface, with deviations indicating the presence of surface structures. With the possibility of sub-nanometer resolution, AFM is particularly adequate to assessments of extracellular vesicles morphology (Witwer et al., 2013). Topography of MVs by AFM from culture supernatants of *L. casei* BL23 and *B. subtilis* 168 is shown in **Figures 4A,B**. AFM phase images reveal MVs with similar morphology for *L. casei* BL23 and *B. subtilis* 168. Even in most cases MVs were observed with a spherical shape, “cup-shape” morphology (with MV center indentation) was also seen between regular MVs. 3D-reconstruction of AFM images confirms that MVs might have a shape ranging from spherical to a “cup-shape” (**Figures 4A,B**). Negative control was also analyzed by AFM, no MVs were observed (**Supplementary Figure S1**).

The values of the edge-to-edge diameter of MVs from *L. casei* BL23 were determined analyzing AFM images. **Figure 4C** shows a histogram with the quantitative analysis of MVs diameter. The mean size for particle distribution by AFM was 33 ± 3 nm for *L. casei* BL23 and 310 ± 5 nm for *B. subtilis* 168 MVs (**Table 1**). The size distribution of *B. subtilis* 168 MVs resulted higher than those obtained by DLS.

#### Transmission Electron Microscopy

**FIGURE 5:**
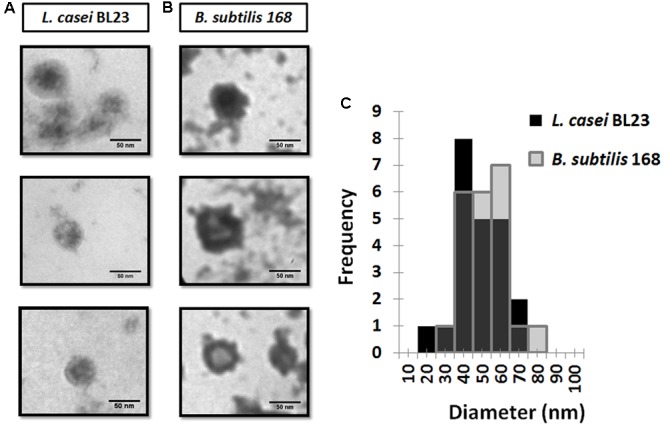
Transmission electron microscopy (TEM) images of MVs from *L. casei* BL23 **(A)** and *B. subtilis* 168 **(B)**. Size-frequency measurements of MVs were expressed as a histogram **(C)**.

To complement the analysis of MVs within the sub-nanometer resolution, we used transmission electron microscopy (TEM) to elucidate details refereed to shape and ultrastructure. TEM images of MVs from culture supernatants of *L. casei* BL23 and *B. subtilis* 168 are shown in **Figures 5A,B**. Electron microscopy examination of *L. casei* BL23 MVs showed a near-spherical shape and a “cup-shape,” (**Figure 5A**) with bilayered membranes and an electron-dense luminal content; which is consistent with the notion that vesicles present bioactive cargo such as proteins or nucleic acids (**Table 1**). However, *B. subtilis* 168 MVs did not always show a central electron-dense core (**Figure 5B**).

The values of the edge-to-edge diameter of MVs were determined analyzing TEM images. From the analysis, only one population with a size of 48 ± 3 nm for *L. casei* BL23 and of 52 ± 3 nm for *B. subtilis* 168 was identified (**Table 1** and **Figure 5C**).

### Formation and Release of MVs

The formation and shedding of MVs was investigated using fluorescence microscopy. CFSE is a fluorogenic dye usually used to label cytoplasmic proteins in living cells. The non-fluorescent dye, when added to live cells, diffuses across the cell membrane where intracellular esterases cleave its acetate group, forming fluorescent CFSE, which is not permeable and is thereby confined to the cytoplasm. This fluorogenic dye reacts to form a covalent bond with lysines and other primary amines resulting in covalently labeled fluorescent proteins in the cellular cytoplasm (Atwal et al., 2016). This protein-specific fluorescent dye is an effective and popular mean to monitor cell division. The capacity of this fluorogenic dye to label cells and MVs with a high fluorescent intensity, combine with its low cell toxicity, make it an excellent dye to measure formation and release of MVs (Nguyen et al., 2016).

**FIGURE 6:**
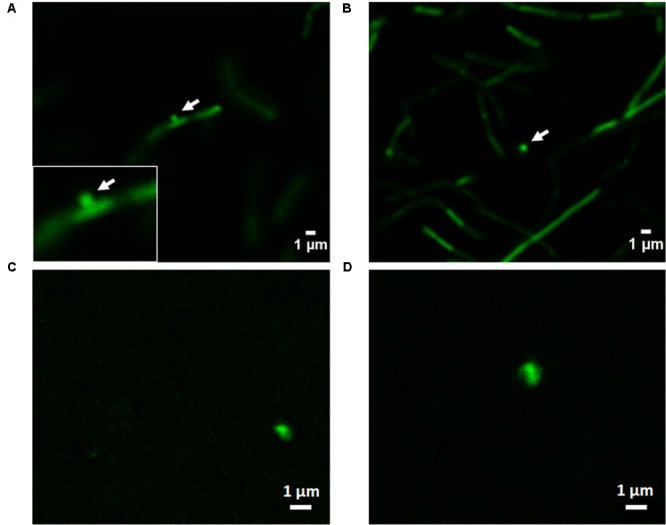
Confocal laser scanning microscopy imaging of the formation of MVs in *L. casei* BL23. **(A,B)** CFSE was used for bacterial staining to investigate the formation and shedding of MVs. **(C,D)** To analyze the aspect of MVs from cell culture supernatant by this technique isolated MVs were stained by CFSE.

Confocal laser scanning microscopy images of *L. casei* BL23 after staining with CFSE are shown in **Figures 6A,B**. The formation and shedding of MVs from the bacterial surface can be clearly visualized in **Figure 6A**. The MVs are formed and released to the extracellular medium (**Figure 6B**). When isolated MVs from cell culture supernatants were stained using CFSE, as expected, they showed an identical signal to that observed in the shedded vesicles (**Figures 6C,D**).

### Chemical Analysis: MVs Contain DNA, RNA and Proteins

*Lactobacillus casei* BL23 derived MVs contained cytoplasmic constituents such as proteins, DNA and RNA (**Table 2**). MVs content was higher in proteins than nucleic acids.

**Table 2:** Microvesicles (MVs) from *L. casei* BL23 contained cytoplasmic constituents such as DNA, RNA and proteins.

	DNA (μg)	RNA (μg)	Proteins (mg)
Mean ± SEM	3.5 ± 1.2	16.3 ± 1.5	0.6 ± 0.2

*Data are mean ± SEM of total DNA, RNA and proteins in MV’s pellet (*n* = 4).*

**FIGURE 7:**
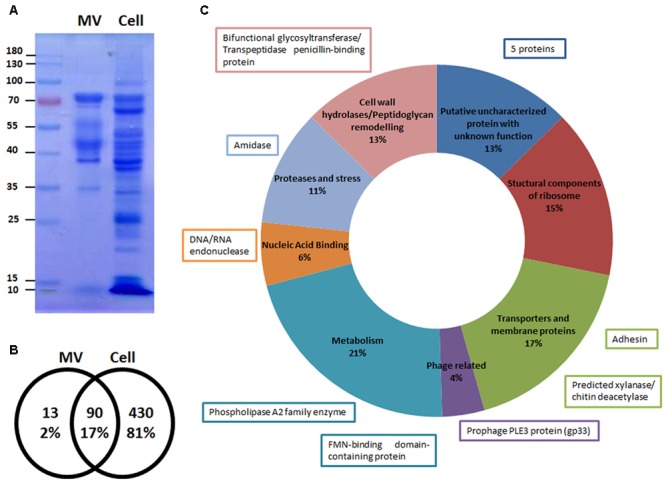
Microvesicles (MVs) are enriched in specific proteins. **(A)** Differential patterns observed between MV and cell extract proteins from *L. casei* BL23 in a Coomassie blue stained SDS-PAGE gel (*n* = 5). **(B)** Venn diagram of the number of proteins present in the MVs and cell extracts identified by LC-MS/MS. **(C)** Proteins of MVs identified by LC-MS/MS were grouped in 8 categories according to its function. The function of the 13 proteins exclusively present in the MVs are shown in boxes.

After SDS-PAGE analysis, different patterns were observed for MVs and whole cell extracts (**Figure 7A**). The most intense bands observed in the lane of the MVs sample correspond to proteins with approximate MWs of 42 and 75 kDa. We hypothesized these proteins could be p40 (LCABL_00230) and p75 (LCABL_02770). The proteomic analysis of the MVs pointed out the presence of these two proteins, that have been widely described in probiotic strains for their role in protection of inflammation and the intestinal epithelium from injury (Van Baarlen et al., 2013). It has been previously described that p40 and p75 of *L. casei* BL23 have cell-wall hydrolase activity and can be located at the bacterial cell surface and/or be secreted to the growth medium (Bäuerl et al., 2011). It is important to note that direct proof about the proteins identity is missing and enzymatic studies should be performed to confirm this hypothesis.

We were able to identify 103 proteins in the MVs; 13 were exclusively present in the MVs and 90 were shared with the whole cell fraction (**Figures 7B,C**). With regard to MVs protein localization: 57% were cytoplasmic proteins and 43% were cell envelope and secretory proteins (Supplementary Table S1). The cytoplasmic proteins included heat and cold shock proteins, several metabolic enzymes, proteases and structural components of the ribosome. The cell envelope and secretory proteins included membrane transporter proteins and cell wall-associated hydrolases like p40 and p75 (**Figure 7C**).

Specifics adhesins from *L. casei* BL23 have been characterized for their capacity to bind to proteins of the extracellular matrix and components of the mucosal layer (Muñoz-Provencio et al., 2010; Munoz-Provencio and Monedero, 2011). One of the proteins only present in MVs and absent in cell extracts is the product of LCABL_31160, annotated as an adhesion protein. This adhesin shares homology to several adhesion proteins present in *L. casei/paracasei* strains (Smokvina et al., 2013).

In addition, we identified a phage structural protein (PLE3, minor capsid) and two proteins probably involved in phage DNA replication, gp42 of PLE2 and gp33 of PLE3 (Dieterle et al., 2016), the latter only present in the MVs. Interestingly, an uncharacterized protein, yhgE, belonging to the phage infection protein (PIP) family was found in the MVs.

## Discussion

In this work we showed for the first time the production of MVs by *L. casei.* Taking into account on prior reports on MVs production by *L. rhamnosus* and *L. plantarum* (Al-Nedawi et al., 2014; Li et al., 2017), the production of MVs in *Lactobacillus casei* group is a newly recognized aspect within this lactic acid bacteria, which are regular resident of the mammalian gastrointestinal microbiome.

MVs from Gram-positive bacteria are described to be bilayered structures from 10 to 400 nm in diameter. In the present work, *L. casei* BL23 MVs were isolated and characterized from a structural and chemical point of view by several techniques.

The ζ-potential determined for *L. casei* BL23 MVs presented a negative value (-8,7 ± 1,9) similar to those reported for other Gram-positive bacteria, i.e., *B. anthracis* with a ζ-potential of -65.67 ± 4.71 mV (Rivera et al., 2010). The negative surface charge could make the MVs highly soluble by virtue of electrostatic repulsion ensuring colloidal stability, i.e., aggregation and precipitation phenomena would be impeded. In line with other analogous structures, the global surface charge of liposomes is negative, as that of exosomes isolated from eukaryotic cell lines, due to the negatively charged phospholipid membrane (Sokolova et al., 2011; Marimpietri et al., 2013; Maas et al., 2015).

Our results showed that the size of *L. casei* BL23 MVs fell into nanoscale as determined by DLS, AFM and TEM. DLS is a suitable method to study particle size in suspension, calculating the differential size distribution of the MV population. The size of MVs from *L. casei* BL23 resulted similar to those reported for other Gram-positive bacteria measured by DLS, i.e., *Staphylococcus aureus* (peak between 20 and 100 nm) (Lee et al., 2009). MVs produced by *B. subtilis* 168 were used for comparison since these vesicles have been well characterized. Brown et al. (2014) reported two discernible populations of MVs as measured by DLS for *B. subtilis* 168, the first peak fell in the range of 50 nm and the second was between 150 and 250 nm. In this work, *B. subtilis* 168 MVs particles revealed only one population of approximately size between 71 and 245 nm, larger than those of *L. casei* BL23.

The standard practice in DLS assumes that the MVs are spherical, although numerous reports suggested that exosomes have a “cup-shaped” geometry in suspension (Chernyshev et al., 2015; Théry et al., 2009). AFM, has opened new perspectives in biomedical research for the investigation of bioparticles (Parot et al., 2007). This technique has been assiduously applied for the assessment of eukaryotic MVs topography (Yuana et al., 2010; Vajen et al., 2015). MVs showed a spherical shape and “cup-shape” morphology (indent of the vesicles in the center) by AFM. Surface desiccation is required to resolve the sample topography and such a procedure can lead to a shape distortion due to a non-uniform drying front (Chernyshev et al., 2015). 3D-reconstruction of AFM images suggested that *L. casei* BL23 MVs might have a shape ranging from spherical to “cup-shape” morphology. On the other hand, van der Pol et al. (2012) claimed that MV structure could change from spherical to hemispherical or flat, according to the composition of the membrane that can cause artifacts in the interpretation of morphology and size due to adhesion of vesicles to the surface

TEM images allow to make a categorization of MVs according to size and electron density and possibly to infer if they contain or not the same cargo (Brown et al., 2014). We observed *L. casei* BL23 MVs with bilayered membranes and electron-dense luminal contents; consistent with the notion that vesicles present bioactive cargo such as proteins or nucleic acids. Also, using this technique the morphology of extracellular vesicles has been previously reported as “cup-shape” due to adhesion, negative staining and drying. Electron microscopy examination of *L. casei* BL23 MVs showed a near-spherical shape and a “cup-shape” with bilayered membranes. However, if this characteristic is an artifact due to extensive sample preparation, or actually an unique feature for MVs and exosomes remains unknown (Marzesco et al., 2005; Chernyshev et al., 2015).

The size of MVs isolated from human blood plasma was shown to be comparable between DLS, AFM and TEM (György et al., 2011). In the case of *L. casei* BL23, the MVs size obtained from DLS data were consistent with measurements from AFM and TEM images. *L. casei* BL23 MVs size were around 30–50 nm. When analyzed by these same techniques, different diameters were calculated for *B. subtilis* MVs. Chernyshev et al. (2015) claimed that for comparison purposes the same methodology should be employed. Modification during sample preparation for TEM and AFM or a less representative microscopic field for inspection could also explain the observed differences.

The conditions that rule the process of genesis and function of MVs still are poorly understood but nowadays it is known that MVs are constantly produced by bacteria (Pathirana and Kaparakis-Liaskos, 2016). CFSE is a used and effective way to track cell division and MVs shedding (Quah and Parish, 2010; Nguyen et al., 2016). Confocal laser scanning microscopy imaging of *L. casei* BL23 showed a protrusion on cells consistent with the emergence of MVs As described above, *L. casei* BL23 MVs size is around (30–50) nm, below the resolution limit of this technique, therefore we cannot assure that there is only one or more MVs shedding per protrusion point.

There is no unified understanding of the mechanisms underlying MVs formation (Al-Nedawi et al., 2014). The analysis of vesicle cargo on *L. casei* BL23 MVs showed the presence of cytoplasmic constituents such as DNA, RNA and proteins. Some authors hypothesized that MVs could deliver functional RNAs and mediate intercellular communication in a process similar to that described in eukaryotes (Al-Nedawi et al., 2014; Foster et al., 2016) and we do not discard that this could also be the case for *L. casei* in the mammalian digestive tract. Emerging evidence in Gram-negative bacteria suggests that extracellular vesicles contain short RNAs (sRNAs) with the potential to target host mRNA stability and/or function (Koeppen et al., 2016).

Proteomic analysis of MVs disclosed a complex protein composition that included cold and heat shock proteins, several metabolic enzymes, proteases and structural components of the ribosome, membrane transporter proteins and cell wall-associated hydrolases like p40 and p75. Bäuerl et al. (2011) reported the presence of these proteins, p40 andp75, in *L. casei* BL23 and described their cell protective and anti-apoptotic effects on human intestinal epithelial cells. It was also shown that p40 of *L. rhamnosus* GG, stimulates the phosphorylation of the epidermal growth factor receptor *in vitro* and *in vivo* in colon epithelial cells and ameliorates intestinal inflammation in mice (Yan et al., 2011; Yoda et al., 2014). In accordance with this, He et al. (2017) recently showed that the culture supernatant from *L. rhamnosus* GG enhances resistance to systemic *Escherichia coli* K1 infection by an increment of intestinal defense in neonates. Although, the authors did not make a direct mention of MVs. They claimed that future studies aimed to identify the active compounds and structural components of the supernatant exerting this effect would be helpful to allow a better understanding of the machinery underlying the positive effect of probiotics. Therefore, MVs could play a key role in these beneficial effects acting either directly or as a delivery system of active compounds.

Bacterial have developed numerous strategies to colonize host mucosae, including modulation of expression of cell surface adhesins. These proteins allow the bacteria to anchor to the human gastrointestinal mucins (Ringot-Destrez et al., 2017). One of the proteins only present in MVs and absent in cell extracts is the product of LCABL_31160, annotated as an adhesion protein. The presence of this protein in MVs suggests that may play an important role in the bacteria-gastrointestinal cells interface. Additionally, p40 and p75 bind to mucins and to intestinal epithelial cells in mouse intestine *ex vivo* (Bäuerl et al., 2011).

Phages are the most abundant biological entities in our gut and are largely unexplored. Mirzaei and Maurice (2017) describe that phages could regulate bacterial communities and thus human health. Recently, we have described three prophages in the genome of *L. casei* BL23 and we demonstrated the induction of two of them (PLE2 and PLE3) after mitomycin C addition (Dieterle et al., 2016). In this work, we reported the presence in MVs of three proteins encoded in these prophages. Particularly gp33 of PLE3 was present exclusively in the MVs. Whether the presence of phage related proteins in the MVs can interfere or promote phage replication should be further studied. The presence of a protein belonging to the PIP family is in agreement with a recent report by Tzipilevich et al. (2016) showing that MVs containing phage receptors could confer transitory sensitivity to phage resistant neighboring bacteria (including non-host species) providing a new route for horizontal gene transfer.

Even though still unexplored, the ability of bacteria to selectively control the MVs content could be exploited for its potential application as delivery systems. This application keeps an enormous unlocked potential since MVs from pathogens have the advantage of appear to be highly immunogenic (Prados-Rosales et al., 2014; Trelis et al., 2016). Recently, Li et al., 2017 found that *L. plantarum*-derived extracellular vesicles enhance host immune responses and provide protective effects on hosts. In this context, MVs result an attractive vaccine strategy, since they are a non-replicative alternative of their parent bacterium that could induce both innate and adaptive immunity.

On the other hand, description of MVs production from regular microbiota including their composition and function could be of crucial significance for maintenance of health. *L casei* BL23 MVs content showed particular features. The proteomic analysis of MVs indicates differences with the respective cytoplasmic content. Therefore, the expression and encapsulation of proteins and sRNA into MVs could represent a scientific novelty, with applications in food, nutraceuticals and clinical therapies.

## Author Contributions

AD contributed to concept/design, acquisition of data, data analysis/interpretation and drafting the manuscript. JM and DM contributed to acquisition of data, data analysis/interpretation. FC, MP, and OP contributed to concept/design, data analysis/interpretation, drafting/revising and approval of the manuscript.

## Conflict of Interest Statement

The authors declare that the research was conducted in the absence of any commercial or financial relationships that could be construed as a potential conflict of interest.

## Supplementary Material

FIGURE S1 Atomic force microscopy (AFM) topography of negative control.
