# Supplementary figures and images for: Lactobacillus casei BL23 Produces Microvesicles Carrying Proteins That Have Been Associated with Its Probiotic Effect
Source: Front Microbiol. 2017 Sep 20;8:1783. doi: 10.3389/fmicb.2017.01783 (PMC5611436; doi:10.3389/fmicb.2017.01783)

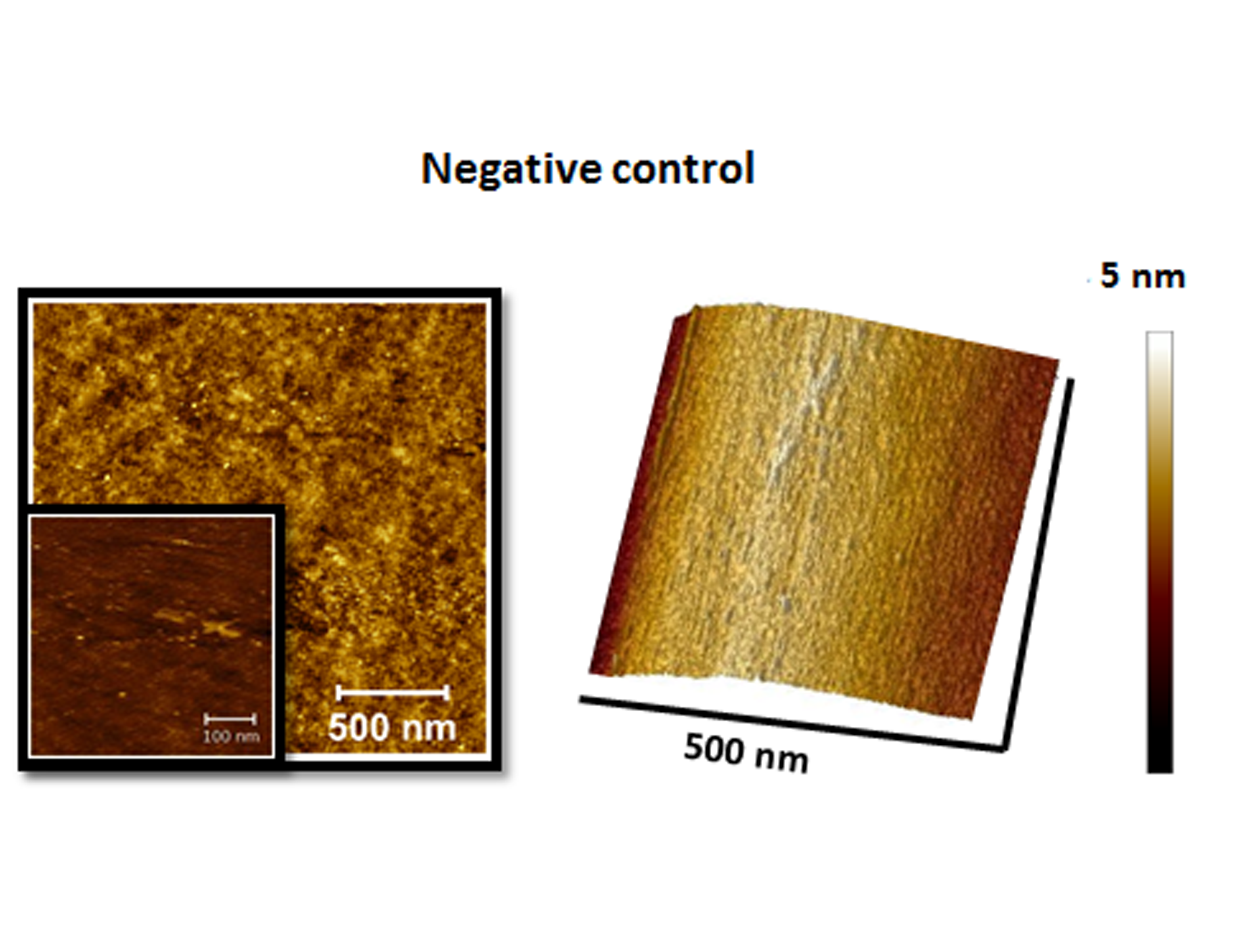

Supplement: FIGURE S1 — Atomic force microscopy (AFM) topography of negative control. [file Image_8.TIF]
