# Supplementary material for: Lactobacillus casei BL23 Produces Microvesicles Carrying Proteins That Have Been Associated with Its Probiotic Effect
Source: Front Microbiol. 2017 Sep 20;8:1783. doi: 10.3389/fmicb.2017.01783 (PMC5611436; doi:10.3389/fmicb.2017.01783)
Supplement: Supplementary file 2 [file Table_1.DOCX]

**Supplementary table.** LC-MC of MVs content.

| **Accession** | **Description** | **Localization** | **Function** | **MW [kDa]** | **Coverage ^a^** | **# PSMs ^b^** | **# Unique Peptides ^c^** |
| --- | --- | --- | --- | --- | --- | --- | --- |
| B3W8J1 | Cell wall-associated hydrolase OS=Lactobacillus casei (strain BL23) GN=LCABL_02480 PE=4 SV=1 | Cell envelope and secretory proteins | Cell wall hydrolases/ Peptidoglycan remodelling | 23 | 64 | 37 | 8 |
| B3W9V6 | Hydrolase protein OS=Lactobacillus casei (strain BL23) GN=LCABL_24090 PE=4 SV=1 | Cell envelope and secretory proteins | Cell wall hydrolases/ Peptidoglycan remodelling | 35.3 | 53 | 55 | 11 |
| B3W994 | Peptidoglycan lytic protein P45 OS=Lactobacillus casei (strain BL23) GN=spl PE=4 SV=1 | Cell envelope and secretory proteins | Cell wall hydrolases/ Peptidoglycan remodelling | 41.5 | 36 | 81 | 18 |
| B3WCW4 | Glyceraldehyde 3-phosphate dehydrogenase OS=Lactobacillus casei (strain BL23) GN=gap-1 PE=3 SV=1 | Cytoplasmatic | Metabolism | 36.7 | 57 | 49 | 17 |
| B3WAW2 | Predicted secreted protein OS=Lactobacillus casei (strain BL23) GN=LCABL_27800 PE=4 SV=1 | Cell envelope and secretory proteins | Cell wall hydrolases/ Peptidoglycan remodelling | 31.4 | 35 | 37 | 7 |
| B3WEN1 | N-acetylmuramoyl-L-alanine amidase, family 3 OS=Lactobacillus casei (strain BL23) GN=ami PE=4 SV=1 | Cell envelope and secretory proteins | Cell wall hydrolases/ Peptidoglycan remodelling | 47 | 39 | 32 | 13 |
| B3W8N5 | Glycoside hydrolase OS=Lactobacillus casei (strain BL23) GN=LCABL_02770 PE=4 SV=1 | Cell envelope and secretory proteins | Cell wall hydrolases/ Peptidoglycan remodelling | 49.7 | 26 | 25 | 8 |
| B3WEV1 | Oligopeptide ABC trasporter substrate binding protein OS=Lactobacillus casei (strain BL23) GN=oppA PE=4 SV=1 | Cell envelope and secretory proteins | Transporters and membrane proteins | 66.4 | 25 | 26 | 12 |
| B3W6S8 | CHAP domain-containing protein OS=Lactobacillus casei (strain BL23) GN=LCABL_00230 PE=4 SV=1 | Cell envelope and secretory proteins | Cell wall hydrolases/ Peptidoglycan remodelling | 42.4 | 32 | 24 | 9 |
| B3WDG2 | Lactobacillus phage PLE3 minor capsid protein E OS=Lactobacillus casei (strain BL23) GN=LCABL_13300 PE=4 SV=1 | Cytoplasmatic | Phage related | 38.3 | 55 | 17 | 11 |
| B3WBI5 | Putative uncharacterized protein OS=Lactobacillus casei (strain BL23) GN=LCABL_30040 PE=4 SV=1 | Cytoplasmatic | Putative uncharacterized proteins with unknown function | 41.3 | 39 | 15 | 7 |
| B3WBI9 | Periplasmic trypsin-like serine protease, HtrA/DegQ family OS=Lactobacillus casei (strain BL23) GN=htrA PE=4 SV=1 | Cell envelope and secretory proteins | Proteases and stress | 44.9 | 22 | 18 | 7 |
| B3W981 | Putative uncharacterized protein OS=Lactobacillus casei (strain BL23) GN=LCABL_21830 PE=4 SV=1 | Cell envelope and secretory proteins | Putative uncharacterized proteins with unknown function | 30 | 30 | 13 | 5 |
| B3W8G9 | Collagen binding protein OS=Lactobacillus casei (strain BL23) GN=cnb PE=3 SV=1 | Cell envelope and secretory proteins | Transporters and membrane proteins | 29.3 | 47 | 14 | 10 |
| B3WE81 | Histone-like DNA-binding protein HU OS=Lactobacillus casei (strain BL23) GN=hbsU PE=3 SV=1 | Cytoplasmatic | Nucleic Acid Binding | 9.5 | 62 | 11 | 5 |
| B3W8Q9 | Inulosucrase OS=Lactobacillus casei (strain BL23) GN=islA PE=4 SV=1 | Cytoplasmatic | Metabolism | 100.5 | 9 | 12 | 5 |
| B3W8H1 | Putative uncharacterized protein OS=Lactobacillus casei (strain BL23) GN=LCABL_06650 PE=4 SV=1 | Cell envelope and secretory proteins | Putative uncharacterized proteins with unknown function | 31.7 | 14 | 10 | 3 |
| B3WCW7 | Enolase OS=Lactobacillus casei (strain BL23) GN=eno PE=3 SV=1 | Cytoplasmatic | Metabolism | 47.1 | 12 | 8 | 3 |
| B3WCW5 | Phosphoglycerate kinase OS=Lactobacillus casei (strain BL23) GN=pgk PE=3 SV=1 | Cytoplasmatic | Metabolism | 42.2 | 22 | 10 | 7 |
| B3WA15 | Cobalamin ECF transporter OS=Lactobacillus casei (strain BL23) GN=LCABL_24680 PE=4 SV=1 | Cell envelope and secretory proteins | Transporters and membrane proteins | 14 | 13 | 10 | 2 |
| B3WAN4 | Sucrose permease (CscB) OS=Lactobacillus casei (strain BL23) GN=LCABL_27020 PE=4 SV=1 | Cell envelope and secretory proteins | Transporters and membrane proteins | 31.2 | 31 | 7 | 5 |
| B3W739 | NADH peroxidase OS=Lactobacillus casei (strain BL23) GN=npr PE=4 SV=1 | Cytoplasmatic | Metabolism | 49.8 | 12 | 8 | 4 |
| B3WE33 | 30S ribosomal protein S20 OS=Lactobacillus casei (strain BL23) GN=rpsT PE=3 SV=1 | Cytoplasmatic | Stuctural components of ribosome | 9.3 | 18 | 7 | 1 |
| B3WBI2 | Putative uncharacterized protein OS=Lactobacillus casei (strain BL23) GN=LCABL_30010 PE=4 SV=1 | Cytoplasmatic | Putative uncharacterized proteins with unknown function | 29.2 | 14 | 6 | 3 |
| B3WDI5 | Putative uncharacterized protein OS=Lactobacillus casei (strain BL23) GN=LCABL_13530 PE=4 SV=1 | Cytoplasmatic | Putative uncharacterized proteins with unknown function | 25.7 | 25 | 5 | 3 |
| B3W7J7 | Membrane protein YhgE, phage infection protein (PIP) family. OS=Lactobacillus casei (strain BL23) GN=yhgE PE=4 SV=1 | Cell envelope and secretory proteins | Phage related | 96.9 | 9 | 7 | 4 |
| B3WA61 | Cysteine aminopeptidase C2 (Bleomycin hydrolase) OS=Lactobacillus casei (strain BL23) GN=pepC2 PE=4 SV=1 | Cytoplasmatic | Proteases and stress | 50.6 | 12 | 6 | 4 |
| B3WCY8 | ABC-type sugar transport system, periplasmic component OS=Lactobacillus casei (strain BL23) GN=LCABL_11540 PE=4 SV=1 | Cell envelope and secretory proteins | Transporters and membrane proteins | 48 | 15 | 6 | 5 |
| B3WAP8 | L-lactate dehydrogenase OS=Lactobacillus casei (strain BL23) GN=ldh PE=3 SV=1 | Cytoplasmatic | Metabolism | 35.5 | 11 | 5 | 3 |
| B3WA01 | 50S ribosomal protein L7/L12 OS=Lactobacillus casei (strain BL23) GN=rplL PE=3 SV=1 | Cytoplasmatic | Stuctural components of ribosome | 12.5 | 33 | 6 | 3 |
| B3WBU6 | Adhesin OS=Lactobacillus casei (strain BL23) GN=LCABL_31160 PE=4 SV=1 | Cell envelope and secretory proteins | Transporters and membrane proteins | 134.3 | 5 | 5 | 4 |
| B3WEY9 | 50S ribosomal protein L27 OS=Lactobacillus casei (strain BL23) GN=rpmA PE=3 SV=1 | Cytoplasmatic | Stuctural components of ribosome | 10.4 | 23 | 5 | 2 |
| B3WAP9 | L,D-transpeptidase/peptidoglycan binding protein OS=Lactobacillus casei (strain BL23) GN=LCABL_27170 PE=4 SV=1 | Cell envelope and secretory proteins | Cell wall hydrolases/ Peptidoglycan remodelling | 50.4 | 10 | 4 | 3 |
| B3W9D4 | Acyltransferase 3 OS=Lactobacillus casei (strain BL23) GN=LCABL_22360 PE=4 SV=1 | Cytoplasmatic | Metabolism | 26.3 | 19 | 5 | 3 |
| B3W8R1 | Pyruvate oxidase OS=Lactobacillus casei (strain BL23) GN=poxL PE=3 SV=1 | Cytoplasmatic | Metabolism | 63.9 | 11 | 4 | 4 |
| B3W8W8 | 50S ribosomal protein L33 OS=Lactobacillus casei (strain BL23) GN=rpmG PE=3 SV=1 | Cytoplasmatic | Stuctural components of ribosome | 5.9 | 27 | 4 | 1 |
| B3W9E4 | Oligopeptide ABC transporter, substrate-binding lipoprotein OS=Lactobacillus casei (strain BL23) GN=oppA PE=4 SV=1 | Cell envelope and secretory proteins | Transporters and membrane proteins | 59.5 | 11 | 4 | 3 |
| B3W9D3 | Wzd OS=Lactobacillus casei (strain BL23) GN=wzd PE=4 SV=1 | Cell envelope and secretory proteins | Cell wall hydrolases/ Peptidoglycan remodelling | 34.8 | 6 | 4 | 2 |
| B3WAK9 | 50S ribosomal protein L29 OS=Lactobacillus casei (strain BL23) GN=rpmC PE=3 SV=1 | Cytoplasmatic | Stuctural components of ribosome | 7.9 | 19 | 3 | 1 |
| B3WE58 | 50S ribosomal protein L32 OS=Lactobacillus casei (strain BL23) GN=rpmF PE=3 SV=1 | Cytoplasmatic | Stuctural components of ribosome | 7.1 | 25 | 3 | 1 |
| B3W9B2 | Wzr OS=Lactobacillus casei (strain BL23) GN=wzr PE=4 SV=1 | Cytoplasmatic | Nucleic Acid Binding | 32.6 | 13 | 3 | 2 |
| B3W9W8 | 10 kDa chaperonin OS=Lactobacillus casei (strain BL23) GN=groS PE=3 SV=1 | Cytoplasmatic | Proteases and stress | 10 | 25 | 3 | 2 |
| B3W979 | RmlC OS=Lactobacillus casei (strain BL23) GN=rmlC PE=4 SV=1 | Cytoplasmatic | Metabolism | 21.5 | 12 | 4 | 2 |
| B3WDC0 | Glucose-6-phosphate isomerase OS=Lactobacillus casei (strain BL23) GN=pgi PE=3 SV=1 | Cytoplasmatic | Metabolism | 49.3 | 7 | 4 | 2 |
| B3WAU5 | Tagatose 1,6-diphosphate aldolase OS=Lactobacillus casei (strain BL23) GN=lacD PE=3 SV=1 | Cytoplasmatic | Metabolism | 36.3 | 15 | 4 | 4 |
| B3WEZ9 | Aluminum resistance protein OS=Lactobacillus casei (strain BL23) GN=Alu-2 PE=4 SV=1 | Cytoplasmatic | Metabolism | 44.6 | 7 | 3 | 2 |
| B3WBZ2 | ABC-type uncharacterized transport system, periplasmic component OS=Lactobacillus casei (strain BL23) GN=LCABL_07680 PE=4 SV=1 | Cell envelope and secretory proteins | Transporters and membrane proteins | 34.1 | 12 | 3 | 3 |
| B3W9W7 | 60 kDa chaperonin OS=Lactobacillus casei (strain BL23) GN=groL PE=3 SV=1 | Cytoplasmatic | Proteases and stress | 57.4 | 8 | 3 | 3 |
| B3W8D3 | Putative uncharacterized protein OS=Lactobacillus casei (strain BL23) GN=LCABL_06730 PE=4 SV=1 | Cell envelope and secretory proteins | Putative uncharacterized proteins with unknown function | 24.2 | 17 | 3 | 2 |
| B3W8K2 | Serine-type D-Ala-D-Ala carboxypeptidase OS=Lactobacillus casei (strain BL23) GN=dacA PE=3 SV=1 | Cell envelope and secretory proteins | Cell wall hydrolases/ Peptidoglycan remodelling | 46.9 | 3 | 3 | 1 |
| B3WF70 | Foldase protein PrsA OS=Lactobacillus casei (strain BL23) GN=prtM PE=1 SV=1 | Cytoplasmatic | Proteases and stress | 33.5 | 3 | 3 | 1 |
| B3W9L3 | 2,3-bisphosphoglycerate-dependent phosphoglycerate mutase OS=Lactobacillus casei (strain BL23) GN=gpmA2 PE=3 SV=1 | Cytoplasmatic | Metabolism | 25.9 | 11 | 2 | 2 |
| B3W8I9 | Transcriptional regulator OS=Lactobacillus casei (strain BL23) GN=lytR PE=4 SV=1 | Cytoplasmatic | Nucleic Acid Binding | 40.1 | 10 | 2 | 2 |
| B3W8P4 | Beta-N-acetylglucosaminidase OS=Lactobacillus casei (strain BL23) GN=glcNAcase PE=4 SV=1 | Cell envelope and secretory proteins | Cell wall hydrolases/ Peptidoglycan remodelling | 61.8 | 2 | 2 | 1 |
| B3W976 | Glycosyhydrolase OS=Lactobacillus casei (strain BL23) GN=LCABL_21780 PE=4 SV=1 | Cell envelope and secretory proteins | Proteases and stress | 38.7 | 9 | 2 | 2 |
| B3WAJ9 | 50S ribosomal protein L15 OS=Lactobacillus casei (strain BL23) GN=rplO PE=3 SV=1 | Cytoplasmatic | Stuctural components of ribosome | 15.5 | 21 | 2 | 2 |
| B3WAL8 | 30S ribosomal protein S10 OS=Lactobacillus casei (strain BL23) GN=rpsJ PE=3 SV=1 | Cytoplasmatic | Stuctural components of ribosome | 11.7 | 25 | 2 | 2 |
| B3WD92 | Predicted Zn-dependent protease OS=Lactobacillus casei (strain BL23) GN=LCABL_12590 PE=4 SV=1 | Cytoplasmatic | Proteases and stress | 30.6 | 5 | 2 | 1 |
| B3WDI4 | DNA/RNA endonuclease OS=Lactobacillus casei (strain BL23) GN=laaO PE=4 SV=1 | Cytoplasmatic | Nucleic Acid Binding | 31.1 | 5 | 2 | 1 |
| B3W8V6 | Putative uncharacterized protein OS=Lactobacillus casei (strain BL23) GN=LCABL_20510 PE=4 SV=1 | Cytoplasmatic | Putative uncharacterized proteins with unknown function | 18.6 | 16 | 2 | 2 |
| B3WEL7 | 30S ribosomal protein S21 OS=Lactobacillus casei (strain BL23) GN=rpsU PE=3 SV=1 | Cytoplasmatic | Stuctural components of ribosome | 6.9 | 14 | 2 | 1 |
| B3W7V2 | Fructose-bisphosphate aldolase OS=Lactobacillus casei (strain BL23) GN=fba PE=4 SV=1 | Cytoplasmatic | Metabolism | 31.5 | 14 | 2 | 2 |
| B3W7K2 | Oligopeptide ABC transporter, substrate-binding lipoprotein OS=Lactobacillus casei (strain BL23) GN=oppA PE=4 SV=1 | Cell envelope and secretory proteins | Transporters and membrane proteins | 59.6 | 5 | 2 | 1 |
| B3WBM4 | Extracellular solute-binding protein, family 1 OS=Lactobacillus casei (strain BL23) GN=LCABL_30430 PE=4 SV=1 | Cell envelope and secretory proteins | Transporters and membrane proteins | 46.5 | 5 | 2 | 2 |
| B3WAL3 | 30S ribosomal protein S19 OS=Lactobacillus casei (strain BL23) GN=rpsS PE=3 SV=1 | Cytoplasmatic | Stuctural components of ribosome | 10.6 | 9 | 2 | 1 |
| B3W7F5 | Phospholipase A2 family enzyme OS=Lactobacillus casei (strain BL23) GN=LCABL_01070 PE=4 SV=1 | Cytoplasmatic | Metabolism | 19.1 | 6 | 2 | 1 |
| B3WE45 | Glutamine ABC transporter, substrate binding and permease protein OS=Lactobacillus casei (strain BL23) GN=glnPH2 PE=4 SV=1 | Cell envelope and secretory proteins | Transporters and membrane proteins | 52.7 | 5 | 2 | 2 |
| B3WAX1 | LemA OS=Lactobacillus casei (strain BL23) GN=LCABL_27890 PE=4 SV=1 | Cell envelope and secretory proteins | Putative uncharacterized proteins with unknown function | 22 | 5 | 2 | 1 |
| B3WA24 | FMN-binding domain-containing protein OS=Lactobacillus casei (strain BL23) GN=cad PE=4 SV=1 | Cell envelope and secretory proteins | Metabolism | 32.7 | 5 | 1 | 1 |
| B3W7K9 | Putative uncharacterized protein OS=Lactobacillus casei (strain BL23) GN=LCABL_01760 PE=4 SV=1 | Cytoplasmatic | Putative uncharacterized proteins with unknown function | 23.6 | 10 | 1 | 1 |
| B3WAJ1 | 50S ribosomal protein L17 OS=Lactobacillus casei (strain BL23) GN=rplQ PE=3 SV=1 | Cytoplasmatic | Stuctural components of ribosome | 14.2 | 9 | 1 | 1 |
| B3WA08 | 50S ribosomal protein L1 OS=Lactobacillus casei (strain BL23) GN=rplA PE=3 SV=1 | Cytoplasmatic | Stuctural components of ribosome | 24.5 | 5 | 1 | 1 |
| B3WDC8 | Prophage Lp2 protein (gp33) OS=Lactobacillus casei (strain BL23) GN=LCABL_12950 PE=4 SV=1 | Cytoplasmatic | Phage related | 12.3 | 15 | 1 | 1 |
| B3WEI8 | Aspartate aminotransferase OS=Lactobacillus casei (strain BL23) GN=aspB PE=3 SV=1 | Cytoplasmatic | Metabolism | 42.5 | 3 | 1 | 1 |
| B3W7T2 | Probable penicillin acylase OS=Lactobacillus casei (strain BL23) GN=pac PE=4 SV=1 | Cell envelope and secretory proteins | Cell wall hydrolases/ Peptidoglycan remodelling | 37.3 | 5 | 1 | 1 |
| B3W8C8 | Putative uncharacterized protein OS=Lactobacillus casei (strain BL23) GN=LCABL_06680 PE=4 B3WAH7SV=1 | Cell envelope and secretory proteins | Putative uncharacterized proteins with unknown function | 22.5 | 9 | 1 | 1 |
| B3WDM9 | Lipoprotein OS=Lactobacillus casei (strain BL23) GN=plpB PE=3 SV=1 | Cell envelope and secretory proteins | Transporters and membrane proteins | 30.4 | 4 | 1 | 1 |
| B3WEI3 | Bifunctional glycosyltransferase/transpeptidase penicillin-binding protein 1A OS=Lactobacillus casei (strain BL23) GN=pbp1A PE=4 SV=1 | Cell envelope and secretory proteins | Cell wall hydrolases/ Peptidoglycan remodelling | 82.7 | 2 | 1 | 1 |
| B3WBI7 | Small heat shock protein (Small heat shock protein hsp19.5) OS=Lactobacillus casei (strain BL23) GN=hsp3 PE=3 SV=1 | Cytoplasmatic | Proteases and stress | 16.5 | 8 | 1 | 1 |
| B3WBL3 | EIID OS=Lactobacillus casei (strain BL23) GN=manN PE=4 SV=1 | Cell envelope and secretory proteins | Transporters and membrane proteins | 33.5 | 4 | 1 | 1 |
| B3W6Y5 | Cold shock protein A OS=Lactobacillus casei (strain BL23) GN=cspA PE=3 SV=1 | Cytoplasmatic | Proteases and stress | 7.2 | 39 | 1 | 1 |
| B3W7F2 | Putative uncharacterized protein OS=Lactobacillus casei (strain BL23) GN=LCABL_01040 PE=4 SV=1 | Cell envelope and secretory proteins | Putative uncharacterized proteins with unknown function | 13.7 | 11 | 1 | 1 |
| B3WAL2 | 50S ribosomal protein L22 OS=Lactobacillus casei (strain BL23) GN=rplV PE=3 SV=1 | Cytoplasmatic | Stuctural components of ribosome | 12.7 | 14 | 1 | 1 |
| B3WAH7 | Putative uncharacterized protein OS=Lactobacillus casei (strain BL23) GN=LCABL_26300 PE=4 SV=1 | Cell envelope and secretory proteins | Putative uncharacterized proteins with unknown function | 32.7 | 3 | 1 | 1 |
| B3WAI4 | Putative uncharacterized protein OS=Lactobacillus casei (strain BL23) GN=LCABL_26370 PE=4 SV=1 | Cytoplasmatic | Putative uncharacterized proteins with unknown function | 8.5 | 16 | 1 | 1 |
| B3W9G2 | Putative uncharacterized protein OS=Lactobacillus casei (strain BL23) GN=LCABL_22640 PE=4 SV=1 | Cell envelope and secretory proteins | Putative uncharacterized proteins with unknown function | 20.2 | 8 | 1 | 1 |
| B3WAV1 | Methionine--tRNA ligase OS=Lactobacillus casei (strain BL23) GN=metS PE=3 SV=1 | Cytoplasmatic | Nucleic Acid Binding | 74.9 | 2 | 1 | 1 |
| B3W8N8 | NADH oxidase OS=Lactobacillus casei (strain BL23) GN=nox PE=4 SV=1 | Cytoplasmatic | Metabolism | 49 | 2 | 1 | 1 |
| B3WAJ6 | Translation initiation factor IF-1 OS=Lactobacillus casei (strain BL23) GN=infA PE=3 SV=1 | Cytoplasmatic | Nucleic Acid Binding | 8.1 | 17 | 1 | 1 |
| B3WD08 | Spermidine/putrescine ABC transporter, substrate binding protein OS=Lactobacillus casei (strain BL23) GN=potD PE=4 SV=1 | Cell envelope and secretory proteins | Transporters and membrane proteins | 40.7 | 4 | 1 | 1 |
| B3W7Q5 | Membrane alanine aminopeptidase OS=Lactobacillus casei (strain BL23) GN=pepN PE=4 SV=1 | Cell envelope and secretory proteins | Proteases and stress | 94.4 | 2 | 1 | 1 |
| B3WAK2 | 50S ribosomal protein L18 OS=Lactobacillus casei (strain BL23) GN=rplR PE=3 SV=1 | Cytoplasmatic | Stuctural components of ribosome | 13 | 8 | 1 | 1 |
| B3WCZ6 | Phosphate acetyltransferase (Phosphotransacetylase) OS=Lactobacillus casei (strain BL23) GN=pta PE=4 SV=1 | Cytoplasmatic | Metabolism | 34.9 | 3 | 1 | 1 |
| B3WCP8 | Phosphoglucomutase OS=Lactobacillus casei (strain BL23) GN=pgm PE=3 SV=1 | Cytoplasmatic | Metabolism | 63.5 | 2 | 1 | 1 |
| B3WAM4 | 30S ribosomal protein S12 OS=Lactobacillus casei (strain BL23) GN=rpsL PE=3 SV=1 | Cytoplasmatic | Stuctural components of ribosome | 15.2 | 8 | 1 | 1 |
| B3WBN4 | Glutamine-binding protein / Glutamine transport system permease protein OS=Lactobacillus casei (strain BL23) GN=glnP PE=4 SV=1 | Cell envelope and secretory proteins | Transporters and membrane proteins | 52.8 | 2 | 1 | 1 |
| B3WE65 | Pyruvate kinase OS=Lactobacillus casei (strain BL23) GN=pyk PE=3 SV=1 | Cell envelope and secretory proteins | Transporters and membrane proteins | 62.8 | 3 | 1 | 1 |
| B3WCN5 | Phosphate ABC transporter, substrate-binding lipoprotein OS=Lactobacillus casei (strain BL23) GN=pstS PE=4 SV=1 | Cell envelope and secretory proteins | Transporters and membrane proteins | 30.6 | 5 | 1 | 1 |
| B3WEB7 | Inorganic pyrophosphatase OS=Lactobacillus casei (strain BL23) GN=ppa PE=4 SV=1 | Cytoplasmatic | Metabolism | 33.8 | 5 | 1 | 1 |
| B3WBK8 | Amidase OS=Lactobacillus casei (strain BL23) GN=LCABL_30270 PE=4 SV=1 | Cytoplasmatic | Proteases and stress | 22.8 | 5 | 1 | 1 |
| B3WAL6 | 50S ribosomal protein L4 OS=Lactobacillus casei (strain BL23) GN=rplD PE=3 SV=1 | Cytoplasmatic | Stuctural components of ribosome | 22.4 | 5 | 1 | 1 |
| B3W972 | Predicted xylanase/chitin deacetylase OS=Lactobacillus casei (strain BL23) GN=LCABL_21740 PE=4 SV=1 | Cell envelope and secretory proteins | Transporters and membrane proteins | 36.6 | 4 | 1 | 1 |
| B3WCT1 | Prophage protein (gp42) OS=Lactobacillus casei (strain BL23) GN=LCABL_10970 PE=4 SV=1 | Cytoplasmatic | Phage related | 18.8 | 6 | 1 | 1 |

**^a^ Coverage:** percentage of predicted protein sequence identified in peptides.

**^b^ # PSM:** peptide spectrum matches.

**^c^ # Unique peptides:** peptide sequences that are unique to a protein

The 13 proteins exclusively present in the MVs are highlights.
